# Sporotrichoid Skin Infection Caused by *Nocardia brasiliensis* in a Kidney Transplant Patient

**DOI:** 10.3390/diseases6030068

**Published:** 2018-07-25

**Authors:** Folusakin Ayoade, Pradeep Mada, Andrew Stevenson Joel Chandranesan, Mohammed Alam

**Affiliations:** 1 Miller School of Medicine, University of Miami, Miami, FL 33136, USA; 2 Texas Health Presbyterian Hospital, Dallas, TX 75231, USA; pradeepmada@texashealth.org; 3 Health Science Center, Louisiana State University, Shreveport, LA 71103, USA; ajoelc@lsuhsc.edu (A.S.J.C.); malam@lsuhsc.edu (M.A.)

**Keywords:** *Nocardia brasiliensis*, sporotrichoid, kidney transplant, lymphocutaneous, skin lesions

## Abstract

Prompt and accurate diagnosis of *Nocardia* skin infections is important in immunocompromised hosts, especially transplant patients. The sporotrichoid form, which is otherwise known as the lymphocutaneous form of *Nocardia* skin involvement, can mimic other conditions, including those caused by fungi, mycobacteria, spirochetes, parasites and other bacteria. Delayed or inaccurate diagnosis and treatment of *Nocardia* skin infections in transplant patients could lead to dissemination of disease and other poor outcomes. *Nocardia brasiliensis* is a rare cause of lymphocutaneous nocardiosis in solid organ transplant patients with only two other cases reported to our knowledge. This case describes a middle-aged man, who presented 16 years post kidney transplant. He developed a sporotrichoid lesion on his upper extremity one week after gardening. Ultrasound showed a 35-cm abscess tract on his forearm, which was subsequently drained. *Nocardia brasiliensis* was isolated from pus culture and he was treated successfully with amoxicillin/clavulanate for 6 months. A review of the relevant literature is included.

## 1. Introduction

Sporotrichoid nocardiosis, which is also known as the lymphocutaneous form of nocardiosis, is a distinct type of cutaneous *Nocardia* infection. Sporotrichoid nocardiosis is characterized by the inoculation of the *Nocardia* bacteria into the skin, which subsequently leads to characteristic lesions distributed along lymphatics in a similar pattern to that seen in sporotrichosis. Therefore, it is not unusual to misdiagnose this form of nocardiosis as sporotrichosis and inappropriately treat with antifungal agents [1]. In immunocompromised patients, this may have serious consequences, including causing a delay in making the correct diagnosis and dissemination of infection to other organs. *Nocardia* can cause infection in both immunocompromised and immunocompetent individuals. However, *Nocardia brasiliensis* causing lymphocutaneous skin infections in a solid organ transplant (SOT) recipient is relatively rare with only two other cases reported to our knowledge. This case illustrates one such presentation and includes a review of the relevant literature.

## 2. Case Presentation

Our case was a 53-year-old man who received a deceased donor kidney transplant 16 years ago, which was presumably due to hypertension. He presented with redness, pain and swelling of his left arm. He reported no trauma but was trimming shrubs and plants one week earlier. He also complained of intermittent fever and chills. There was no significant animal contact or recent travels. He took cyclosporine and mycophenolate for immunosuppression but had no recent use of corticosteroids.

**Figure 1:**
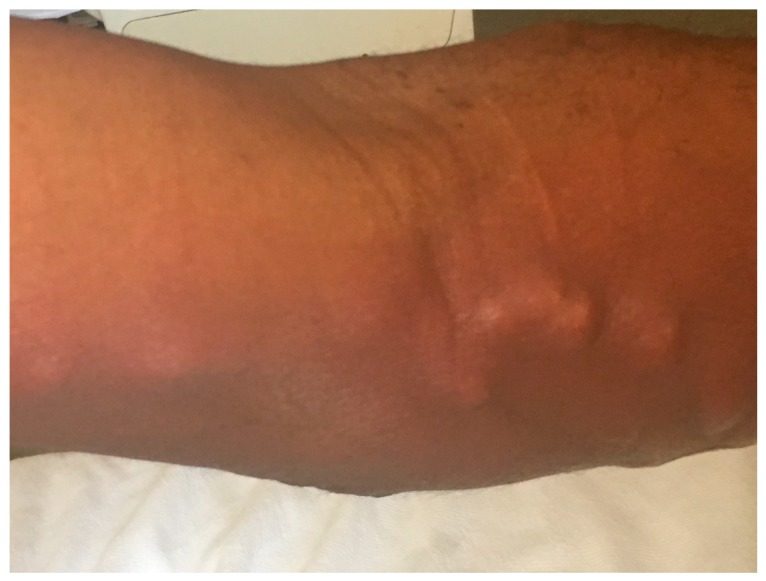
Sporotrichoid lesion on the left arm and forearm.

**Figure 2:**
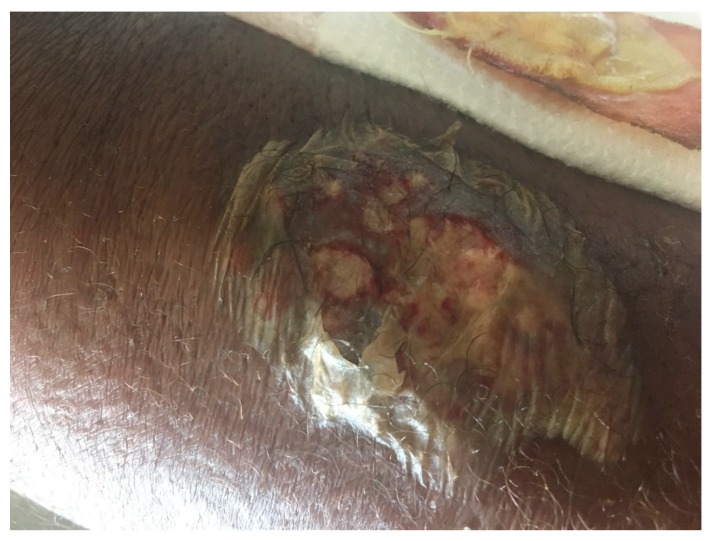
Ulcerative lesion on the left forearm.

Evaluation showed a temperature of 100.3 °F, heart rate of 99 beats/min, respiration of 20 cycles/min and blood pressure of 109/71 mmHg. Skin examination showed erythema, tenderness and swelling on the medial aspect of his left arm and forearm in a linear fashion (Figure 1). There was also a wound on his left forearm, which was located distal to the sporotrichoid lesion, which he thought might have been due to an insect bite or an injury related to his recent gardening (Figure 2). There was an old unused dialysis graft on the forearm, which appeared to be unaffected. There were no significant lymph node enlargement and the examination of the other systems was completely unremarkable.

Ultrasound imaging of the left arm showed hypoechoic, tubular type structures (the largest measuring 8 × 5 mm) in the subcutaneous fat, which is consistent with an abscess. Two views of the chest radiograph was normal.

The patient underwent incision and drainage of a long abscess tract, which measured approximately 35 cm. Frank purulent drainage was expressed from the tract and was sent for bacterial, fungal and mycobacterial cultures. In the meantime, the patient was started on empirical intravenous piperacillin/tazobactam and vancomycin while awaiting culture results. Sporotrichosis was considered as a strong differential but antifungal therapy was deferred until the culture results became available.

Significant laboratory findings include a white blood cell count of 12.2 × 10^9^/L, blood urea nitrogen of 56 mg/dL and creatinine of 2.7 mg/dL from a baseline of 1.8 mg/dL. Blood cultures and culture of the distal left forearm wound were negative for any growth.

**Figure 3:**
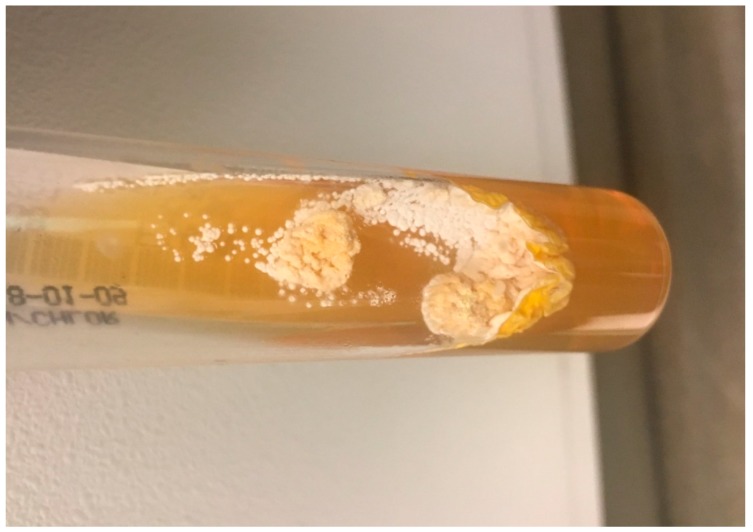
Yellowish white colonies growing on Sabouraud dextrose agar without chloramphenicol identified by 16SrRNA as *Nocardia brasiliensis*.

Pale, yellowish-white colonies were isolated from fungal cultures of the pus on Sabouraud dextrose agar without Chloramphenicol after 11 days (Figure 3).

The gram stain of the colonies revealed aerobic, branching, gram positive rods, which were further identified by 16S ribosomal RNA (16S rRNA) sequencing as *Nocardia brasiliensis*. Susceptibility results showed that the isolate was sensitive to trimethoprim–sulfamethoxazole, linezolid, amoxicillin/clavulanate, moxifloxacin, amikacin, ceftriaxone and tobramycin; had intermediate sensitivity to doxycycline and minocycline; and was resistant to imipenem, ciprofloxacin and clarithromycin. The bacterial cultures did not grow and were discarded after 5–7 days per institution protocol. Mycobacterial cultures were also negative. Pathology of the excised abscess tissue revealed acute inflammation and abscess formation, which involved the dermis and subcutaneous tissue with dermal and fat necrosis. No bacterial or fungal elements were seen on appropriate stains. Due to his worsening kidney function, a renal biopsy of his transplanted kidney was obtained, which showed findings that were consistent with IgA nephritis, chronic allograft nephropathy and glomerulosclerosis.

**Figure 4:**
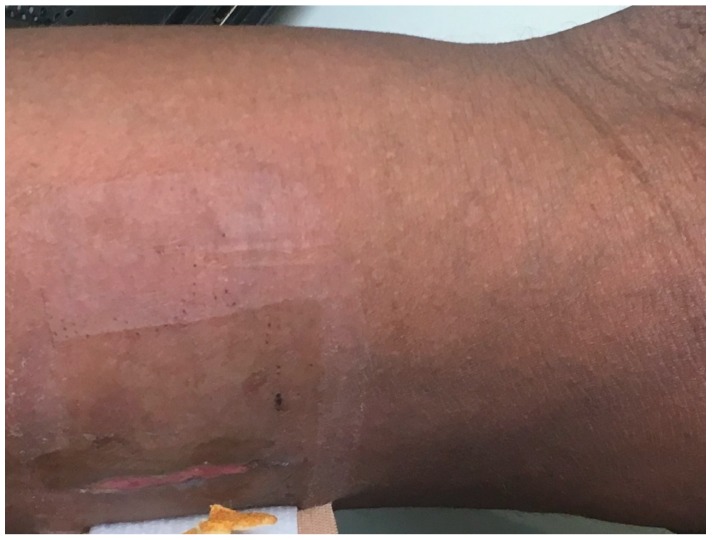
Healing surgical wounds on left forearm four weeks after abscess drainage.

He received amoxicillin/clavulanate 875/125 twice daily for 6 months for definitive treatment of his Nocardia infection. Three weeks post discharge, the sporotrichoid lesion significantly improved and the abscess wounds showed evidence of good healing (Figure 4). At his 6-month follow up, he had made a complete recovery with no residual lesions.

## 3. Discussion

*Nocardia* are bacteria, which are best described as aerobic, gram positive actinomycetes. Nocardiosis, which is the disease associated with *Nocardia*, has varied clinical presentations. It can affect any organ but predominantly involves the lungs, brain and skin. When two or more organs are involved, the disease is described as disseminated. The clinical presentation could mimic several other disease entities, including those caused by bacterial, fungal, parasitic and mycobacterial pathogens. This prompts some to describe the disease as a great imitator [2,3]. In addition to nocardiosis and sporotrichosis, other possible differential diagnoses of sporotrichoid lesions include leprosy, cutaneous leishmaniasis, cutaneous tuberculosis, sporotrichoid mycobacteriosis, thrombophlebitis, tularemia and yaws [1,4].

*Nocardia* typically causes disease in immunocompromised hosts but up to 30% of presentations occur in immunocompetent individuals [5,6,7,8]. *Nocardia* is an important part of the normal soil microflora and are considered environmental pathogens that are found worldwide [9]. There are over 75 species, of which 30 or more are pathogenic [10].

The occurrence rate of nocardiosis (all forms) in SOT is in the range of 0.04–3.5% [11,12,13]. More specifically, in kidney transplant patients, the incidence is in the range of 0.4–1.3% [14,15,16].

The acquisition of cutaneous nocardiosis is generally two-fold: either as direct inoculation following gardening or similar activities; or dissemination to skin from other systemic sources [7,17,18,19]. There is lack of consensus on which of these two forms of cutaneous nocardiosis predominates. Our patient in question likely contracted his infection from his gardening activity and his immunocompromised status likely increased his risk for infection.

Sporotrichoid nocardiosis can spread from one area of the skin to involve other skin areas or become disseminated to other organs [18,20]. However, since lung involvement is the commonest overall presentation of nocardiosis, the inhalation of contaminated soil material is considered to be the primary acquisition mode of most infections [6,15,21].

It is well established that solid organ transplantation is a recognizable risk factor for developing nocardiosis [13,22,23]. It has also been shown that transplant patients who develop nocardiosis have a 10-fold mortality risk at one year compared to matched controls [24]. Specific risk factors for nocardiosis in transplant recipients have been described. In a large series of 5126 organ transplant recipients, Peleg et al. noted that high dose steroids, high dose of calcineurin inhibitors and previous history of cytomegalovirus disease in the preceding 6 months are independent risk factors for the development of *Nocardia* infection [11]. Similarly, Coussement et al. reported patient age, length of stay in the intensive care unit after SOT, use of tacrolimus, high calcineurin inhibitor trough levels in the month before diagnosis and corticosteroid dose at the time of diagnosis as being independently associated risk factors for nocardiosis [12]. The only risk factor identified in our case was age but potentially, his chronic use of anti-rejection immunosuppressive agents probably also has a role to play. The cyclosporine blood level for our patient was not measured during his admission stay and thus, we are unable to determine if high dose or high trough levels of calcineurin inhibitor potentially contributed to his infection risk.

In terms of which organ transplant recipients are more at risk for infection, Peleg et al. found that patients with lung (3.5%) and heart (2.5%) transplants have the highest risk, while patients with kidney (0.2%) and liver (0.1%) transplants have the lowest risk of developing *Nocardia* infection [11]. Similarly, Santos et al. found that the majority (37%) of the transplant recipients in their series who developed *Nocardia* infections had a previous lung transplant [13].

The cutaneous manifestations of nocardiosis have been well described even though they are generally rare complications following SOT (Naldi et al.) [25]. There are four main patterns:

- Primary Cutaneous: typical, from direct skin inoculation.
- Lymphocutaneous: when lymphatics are involved and characteristic sporotrichoid spread is present.
- Mycetoma: usually an indolent, granulomatous infection, which is mostly located on lower extremities with chronic draining sinuses and ulcers. This is very common in tropical countries.
- Skin deposits from disseminated disease.

Among all *Nocardia* pathogens, *N. brasiliensis* is one of the rarest species that infect both solid organ transplant patients and immunocompetent hosts [11,12,22]. It is very difficult to differentiate *Nocardia* species based on clinical presentation but *N. brasiliensis* appear to be more notorious for causing cutaneous disease when compared to other known pathogenic *Nocardia* species [13,21]. *N. brasiliensis* has also been found in the lymphocutaneous and mycetoma form more frequently compared to other species of *Nocardia* [26,27]. A Taiwan study that analyzed a 10-year data found nodulopustular pyoderma associated with sporotrichoid spreading was the most common skin presentations caused by *N. brasiliensis* [26].

Table 1 contains a summary of cutaneous Nocardia infection that is attributable to N. brasiliensis in SOT recipients based on our extensive literature search.

Different acquisition modes of cutaneous nocardia infection have been described, including motor vehicle injury [28] or puncture wounds related to gardening activities [29,30] but in some cases, the mode of acquisition is unknown [31]. Morphologically, the lesions may be in the form of ulcers, abscesses, granulomas, other soft tissue lesions and lymphocutaneous infection [32].

Pathogenesis of *Nocardia* infection is complex and involves multiple mechanisms, some of which are incompletely understood [9]. The pathogenesis involves different factors, including host defences, immune status, species type and virulence. The defect in cell immunity is considered to play a major role [33]. The full details of nocardial pathogenesis are beyond the scope of this article.

As far as antimicrobial treatment is concerned, trimethoprim–sulfamethoxazole (TS) is the drug of choice and many strains of *Nocardia* have demonstrated susceptibility to TS in several reports, including the isolate in our case. However, we chose an alternate regimen given the patient’s worsening kidney failure in the setting of diminished renal reserve. Apart from TS, other reasonable oral choices based on antibiotic susceptibilities include amoxicillin–clavulanate, minocycline and moxifloxacin [15,34]. It is important to note that while TS is often a preferred treatment option for nocardiosis, several cases of breakthrough infection have been well described in patients on TS prophylaxis [11,12,22,23].

*Nocardia* skin infection in SOT recipients can develop at any time, which can be as early as one month to as late as 192 months post-transplant as typified by our case [5,11,14,15]. Some cases of *Nocardia* infection after 244 months have been reported [12]. It is unclear why our patient in question presented with *Nocardia* infection after many years after his transplant and if other unique factors, such as race, gender or type of immunosuppressive agent, played any role. Although infections that are encountered after 6 months post-transplant are typically community-acquired and mirrors the general population, ongoing impaired cellular immunity by immunosuppressive agents, especially cyclosporine use as in our patient, typically increase infection risk [35]. Focused studies to explain the potential association of possible risk factors for late Nocardia infection in SOT recipients are needed.

In conclusion, *Nocardia* skin infection has varied presentations and can mimic other conditions as alluded to above [4,36]. High index of suspicion, prompt diagnosis and appropriate treatment is imperative, especially in SOT recipients.

## 4. Learning Points

- Sporotrichoid or lymphocutaneous skin infections caused by *Nocardia brasiliensis* are relatively rare in solid organ transplant patients and can mimic several other infectious skin conditions.
- Prompt and accurate diagnosis of nocardial skin infection is important, especially in transplant patients.
- Nocardia culture typically requires a prolonged time to grow but may occasionally be recovered from standard fungal media as in our case.

## Figures and Tables

**Table 1:** Summary of cutaneous *Nocardia* infection attributable to *N. brasiliensis* in solid organ transplant recipients.

	Current Case	Parra et al. [5]	Queipo-Zaragoza et al. [14]	Arduino et al. [15]	García-Benítez et al. [34]	Santos et al. [13]
Organ transplanted	Kidney	Liver	Kidney	Kidney	Kidney	Kidney
Months post-transplant	192	24	1	9.5	NA	1
Immunosuppressive agents	Cyclosporine, Mycophenolate	Tacrolimus, Methylprednisolone	Azathioprine, Cyclosporine, Prednisone	Cyclosporine, Prednisone	Azathioprine, Cyclosporine, Prednisone	NA
Form of cutaneous disease	Lymphocutaneous	Lymphocutaneous	Likely disseminated (from lung)	Disseminated (from lung)	Primary cutaneous	Disseminated
Clinical features	Painful nodular skin lesions 1 week after gardening	Fever, jaundice, painful ulcer, tender nodules and enlarged local lymph nodes	Subcutaneous inflammatory nodules	Skin nodule	Painful draining nodule	Skin lesions. Also involved the intestine and lung
Diagnosis	16 S r RNA of pus	Culture of nodule aspirate	Culture of nodule and bronchial aspirates, and peritoneal fluid	Skin biopsy. No further information available	Skin biopsy culture	Subcutaneous fluid
Antibiotic Susceptibility	TS, L, AC, M, A, C, T	NA	NA	T, G, AC, S and possibly TS, A, CP	NA	NA
Pathology	Acute inflammation, abscess formation in the dermis and subcutaneous tissue with dermal and fat necrosis	Branching hyphae, atrophic epidermis, dermal abscess with neutrophilic infiltration and necrosis	Branching filaments on Ziehl-Neelsen staining	NA	Necrosis and acute inflammation	NA
Treatment	AC for 6 months	TS for 8 weeks	TS, Vancomycin and Cephalosporin	AC for 7 months	I, CE, A for 7 days, then MI	NA
Outcome	Cure	Cure	Death	Cure	Cure	Death

Trimethoprim-sulfamethoxazole (TS), linezolid (L), amoxicillin-clavulanate (AC), moxifloxacin (M), amikacin (A), ceftriaxone (C) and tobramycin (T), Gentamicin (G), Sulfisoxazole (S), Ciprofloxacin (CP), Imipenem (I), Cefepime (CE), Minocycline (MI), Not available (NA).
